# Extracorporeal Cardiopulmonary Resuscitation With Ventricular Unloading in Fulminant Influenza A (H1N1) Perimyocarditis: A Case Report

**DOI:** 10.1155/crcc/6129145

**Published:** 2026-10-31

**Authors:** Maria Francesca Marchetti, Flavio Frau, Gianfranco De Candia, Emiliano Maria Cirio, Mauro Cadeddu, Michele Porcu, Roberta Montisci

**Affiliations:** ^1^ Clinical Cardiology and Cardiac Intensive Care Unit Department of Medical Sciences and Public Health University Hospital of Cagliari Cagliari Italy; ^2^ Cardiac Anesthesia Unit Department of Cardiothoracic and Vascular Sciences ARNAS G. Brotzu Cagliari Italy; ^3^ Cardiac Surgery Unit Department of Cardiothoracic and Vascular Sciences ARNAS G. Brotzu Cagliari Italy; ^4^ Department of Radiology University Hospital of Cagliari Cagliari Italy

## Abstract

**Background:**

Fulminant perimyocarditis is an uncommon cause of cardiogenic shock and refractory cardiac arrest. We describe Influenza A (H1N1)–associated perimyocarditis managed with a stepwise extracorporeal resuscitation strategy.

**Case:**

A 54‐year‐old woman presented with chest pain, hypoxemia, and cardiogenic shock. Electrocardiography (ECG) showed diffuse ST‐segment elevation. Echocardiography revealed severe biventricular dysfunction and a fibrinous pericardial effusion and coronary angiography excluded obstructive disease. Despite vasoactive support and mechanical circulatory support with microaxial flow pump Impella CP (flow 2.7 L/min.), she deteriorated to Society for Cardiovascular Angiography and Interventions (SCAI) Stage E with pulseless electrical activity (PEA). During automated chest compressions, mechanical circulatory support with femoral Impella CP was rapidly escalated to venoarterial extracorporeal membrane oxygenation (VA‐ECMO). Respiratory polymerase chain reaction (PCR) was positive for Influenza A (H1N1) virus. Antiviral therapy, heparin anticoagulation, ventilation, and organ support were instituted. Hemodynamics stabilized, lactate cleared, and neurological examination after decannulation was intact. Progressive myocardial recovery permitted VA‐ECMO explant on Day 3 and Impella CP removal 24 h thereafter. She was extubated on Day 6 and discharged from intensive care unit (ICU) on Day 10. Cardiac magnetic resonance (CMR) imaging confirmed myocarditis; echocardiography at follow‐up showed preserved biventricular function.

**Conclusion:**

In fulminant perimyocarditis with refractory arrest, rapid activation of an extracorporeal cardiopulmonary resuscitation (ECPR) pathway with early VA‐ECMO—combined with ventricular unloading—can bridge to myocardial recovery and favorable neurological outcome. Regional systems enabling safe transfer under continuous cardiopulmonary resuscitation (CPR) are critical.

## 1. Background

Fulminant myocarditis represents a rare but life‐threatening manifestation of myocardial inflammation, characterized by rapid progression to severe biventricular dysfunction, cardiogenic shock, malignant arrhythmias, and multiorgan failure. Viral infections remain the most common etiology, with influenza viruses, including H1N1, frequently implicated. Despite advances in diagnostic and therapeutic strategies, fulminant myocarditis continues to pose significant clinical challenges because of its unpredictable course and high mortality in the absence of timely recognition and intervention [1].

The management of these critically ill patients often requires escalation from pharmacological therapy to advanced mechanical circulatory support (MCS), such as intra‐aortic balloon pump, microaxial pumps, or venoarterial extracorporeal membrane oxygenation (VA‐ECMO). In recent years, the combined use of devices like Impella and VA‐ECMO (the so‐called “ECMELLA” approach) has been increasingly adopted to provide both ventricular unloading and systemic perfusion [2].

Here, we describe the case of a middle‐aged woman with fulminant myocarditis secondary to Influenza A (H1N1) infection, complicated by cardiogenic shock and refractory cardiac arrest. The case illustrates the pivotal role of early MCS and extracorporeal cardiopulmonary resuscitation (ECPR) in achieving full recovery of cardiac function and favorable long‐term outcome.

## 2. Case Presentation

A 54‐year‐old Caucasian woman, with a past medical history of arterial hypertension and hypercholesterolemia under treatment and previous left breast cancer treated surgically and with chemotherapy currently in remission, presented with acute chest pain. She was of normal weight (65 kg, 165 cm), with a body mass index (BMI) of 23.8 kg/m^2^ and reported a flu‐like illness 7 days earlier, characterized by fever and pharyngodynia, which had resolved after 4 days of nonsteroidal anti‐inflammatory therapy (ibuprofen 600 mg three times daily). However, profound asthenia persisted.

Following the onset of typical chest pain the previous evening, the patient activated the emergency medical service. Upon arrival at her home, rescuers documented impaired consciousness with Glasgow Coma Scale (GCS) 11, hypoxemia with peripheral oxygen saturation (SpO_2_) 85% on room air, and severe hypotension with noninvasive blood pressure (NIBP) of 70/40 mmHg. The electrocardiography (ECG) showed sinus rhythm at 120 bpm with diffuse concave ST‐segment elevation in the anterolateral and inferior leads (Figure 1). Oxygen therapy was initiated using a nonrebreather mask with a fraction of inspired oxygen (FiO_2_) of 100%, followed by 750 mL of crystalloid infusion and 25 mg of intravenous ephedrine, with minimal improvement. The ECG was transmitted to the tertiary cardiac intensive care unit (ICU), which advised immediate direct transfer to the catheterization laboratory.

**Figure 1:**
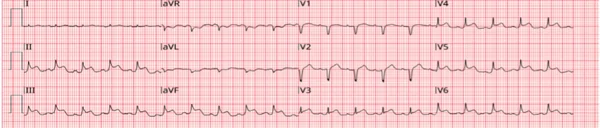
Electrocardiogram at presentation. The 12‐lead electrocardiogram demonstrates sinus tachycardia with diffuse concave ST‐segment elevation across the anterolateral and inferior leads, in the absence of reciprocal changes, a pattern suggestive of acute myocarditis rather than acute coronary syndrome.

On arrival 15 minutes later, the patient′s condition had worsened, with severe cardiogenic shock classified as Society for Cardiovascular Angiography and Interventions (SCAI) Stage C–D: GCS 5, blood pressure 60/30 mmHg, sinus rhythm 120 bpm, mottled skin, and arterial blood gas analysis showing profound metabolic acidosis with lactate level of 10 mmol/L. Echocardiography demonstrated a nondilated left ventricle with mild concentric hypertrophy and severe global systolic dysfunction characterized by diffuse hypokinesia without regional wall motion abnormalities, a left ventricular ejection fraction (LVEF) of 20%, a left ventricular outflow tract velocity‐time integral (LVOT VTI) of 9 cm, and a tissue Doppler S ^′^ velocity of 4 cm/s. Right ventricular function was mildly impaired, with a tricuspid annular plane systolic excursion (TAPSE) of 14 mm, whereas an E/e ^′^ ratio of 21 suggested elevated left ventricular filling pressures. A mild to moderate pericardial effusion with fibrinous component (12 mm) was noted, without tamponade.

The patient was intubated and mechanically ventilated in protective mode. A central venous catheter and arterial line were placed. Despite inotropic (dobutamine 10 *μ*g/kg/min) and vasopressor (norepinephrine 0.1 *μ*g/kg/min) support, shock persisted with a Vasoactive‐Inotropic Score (VIS) of 20. Microaxial flow pump Impella CP was implanted via the femoral artery. The device was set at P6, providing a flow of 2.7 L/min to avoid excessive left ventricular unloading in the setting of mild right ventricular dysfunction, resulting in partial hemodynamic stabilization with a mean arterial pressure of 60 mmHg. Coronary angiography via radial access revealed no coronary obstructive lesions. Right heart catheterization confirmed severe cardiogenic shock, with a cardiac output (CO) of 2.6 L/min, cardiac index (CI) of 1.43 L/min/m^2^, pulmonary capillary wedge pressure (PCWP) of 21 mmHg, and pulmonary artery pulsatility index (PAPi) of 0.35. Laboratory tests showed markedly elevated high‐sensitivity Troponin I (> 25,000 ng/L), creatine phosphokinase (CPK; 2159 U/L), creatine kinase‐MB (CK‐MB; 166 U/L), and N‐terminal pro‐B‐type natriuretic peptide (NT‐proBNP; > 35,000 pg/mL). White blood cell count was 13,580/mm^3^ with neutrophilia and lymphopenia. A multiplex polymerase chain reaction (PCR) respiratory pathogen panel performed on a nasopharyngeal swab was positive for Influenza A (H1N1), whereas all other tested respiratory viral and bacterial pathogens were negative. A separate rapid antigen test for severe acute respiratory syndrome Coronavirus 2 (SARS‐CoV‐2) was negative.

Despite inotropic support and mechanical cardiac assistance with Impella CP, within 100 min of first medical contact, the patient deteriorated further, with right ventricular failure (TAPSE 10 mm), worsening lactate levels, and new‐onset atrial fibrillation at 180 bpm, refractory to electrical cardioversion. This was followed by pulseless electrical activity (PEA) with PAM 25 mmHg. Despite external cardiac massage and repeated adrenaline boluses, return of spontaneous circulation was not achieved after 10 min. A LUCAS 2 mechanical chest compression device was deployed, enabling ongoing advanced resuscitation during transport to the cardiac surgery unit, located 5 km away. There, within 40 minutes of cardiac arrest onset, percutaneous femoral VA‐ECMO was initiated as ECPR.

The patient was admitted to the cardiothoracic ICU under VA‐ECMO support (flow 3.4 L/min, sweep gas 2 L/min, FiO_2_ 75%), receiving sedation with propofol and sufentanil, low‐dose epinephrine and norepinephrine, and continuous heparin anticoagulation with antithrombin supplementation. Antiviral therapy with oseltamivir (75 mg twice daily) was commenced. Neither corticosteroids nor other immunosuppressive therapies were administered. In the following 24 h, catecholamines were discontinued, and levosimendan infusion was introduced. Lactate levels normalized, and acidosis resolved.

On Day 3, echocardiography demonstrated marked recovery of biventricular function (LVEF 50%, LVOT VTI 15 cm, TAPSE 18 mm, S ^′^ 12 cm/s). VA‐ECMO was successfully weaned and explanted, followed by removal of the Impella CP 24 h thereafter. The patient was extubated on Day 6 with preserved neurological status. During the ICU stay, she developed episodes of hemodynamically stable atrial fibrillation, treated with pharmacologic and electrical cardioversion, and a *Klebsiella pneumoniae* pneumonia, which responded to combined antibiotic therapy with piperacillin‐tazobactam and vancomycin.

She was discharged from the cardiothoracic ICU on Day 10 in stable condition and transferred to the referring cardiac ICU. Serial echocardiograms revealed progressively normalization of left ventricular size and function (LVEF 66%, LVOT VTI 21.6 cm), with mild residual diastolic dysfunction (E/e ^′^ 11) and trivial fibrinous pericardial effusion. Cardiac magnetic resonance (CMR) imaging on Day 12 confirmed the diagnosis of fulminant myocarditis, showing patchy intramyocardial late gadolinium enhancement in the basal and midinferior segments, with myocardial edema (Figure 2).

**Figure 2:**
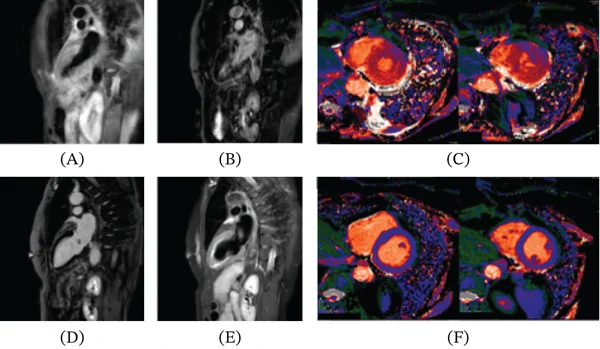
Cardiac magnetic resonance imaging at baseline and follow‐up. (A–C) Baseline imaging demonstrating myocardial inflammation with increased signal intensity on T1‐weighted sequences, patchy intramyocardial late gadolinium enhancement, and elevated T1 mapping values, consistent with diffuse myocardial edema. (D–F) Follow‐up imaging showing resolution of myocardial edema, normalization of T1 values, and marked reduction of late gadolinium enhancement, consistent with myocardial recovery.

The patient was discharged home on Day 32, hemodynamically stable, with normal respiratory and renal function, and only a mild left lower limb weakness related to VA‐ECMO cannulation, which resolved with physiotherapy.

At 6‐month follow‐up, she had returned to full independence, including resumption of work activities. Echocardiography and CMR confirmed complete recovery of biventricular systolic function, resolution of edema, and markedly reduced residual late gadolinium enhancement (Figure 2). She remained on optimized cardiological therapy with excellent quality of life.

The overall clinical course and timing of key interventions are summarized in Figure 3.

**Figure 3:**
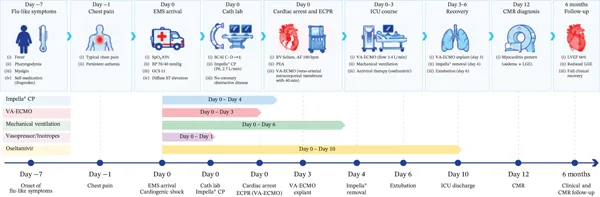
Timeline of the patient′s clinical course, from the onset of flu‐like symptoms to 6‐month follow‐up. The timeline summarizes the progression from cardiogenic shock to cardiac arrest requiring extracorporeal cardiopulmonary resuscitation (ECPR), temporary mechanical circulatory support with Impella CP, and venoarterial extracorporeal membrane oxygenation (VA‐ECMO); the subsequent clinical recovery; and the cardiac magnetic resonance (CMR) findings. The duration of the main therapeutic interventions is also shown. Abbreviations: AF, atrial fibrillation; CMR, cardiac magnetic resonance; ECPR, extracorporeal cardiopulmonary resuscitation; EMS, emergency medical services; GCS, Glasgow Coma Scale; ICU, intensive care unit; LGE, late gadolinium enhancement; LVEF, left ventricular ejection fraction; PEA, pulseless electrical activity; RV, right ventricular; SCAI, Society for Cardiovascular Angiography and Interventions; SpO_2_, peripheral oxygen saturation; VA‐ECMO, venoarterial extracorporeal membrane oxygenation.

## 3. Discussion

Fulminant myocarditis represents one of the most dramatic clinical presentations of acute myocardial inflammation, characterized by rapidly progressive hemodynamic deterioration, severe biventricular dysfunction, and a high risk of refractory cardiogenic shock or cardiac arrest [1]. Although uncommon, its clinical trajectory can be catastrophic, necessitating timely recognition and escalation of circulatory support [2]. The present case illustrates several critical aspects in the management of influenza‐associated fulminant myocarditis, emphasizing both the challenges of diagnosis and the therapeutic implications of advanced MCS.

From a diagnostic perspective, the patient presented with chest pain, electrocardiographic ST‐segment elevation, and biomarker evidence of myocardial injury, closely mimicking an acute coronary syndrome. In such circumstances, early exclusion of obstructive coronary artery disease is crucial as clinical management diverges profoundly between acute coronary syndromes and myocarditis. Coronary angiography confirmed normal epicardial vessels, whereas echocardiography revealed severe biventricular dysfunction, pericardial effusion, and myocardial echogenicity abnormalities, consistent with acute inflammatory myocardial injury. Subsequent CMR confirmed diffuse myocardial edema and late gadolinium enhancement, consolidating the diagnosis of myocarditis. Importantly, PCR testing identified Influenza A (H1N1) infection, supporting a viral etiology [3].

Fulminant myocarditis is a distinct clinicopathological entity compared with acute myocarditis of milder severity. Histopathological studies describe diffuse myocardial necrosis, interstitial edema, and dense inflammatory infiltrates, which typically underlie the precipitous decline in cardiac performance [4]. Although endomyocardial biopsy remains the gold standard for diagnosis, its clinical feasibility is often limited in unstable patients, and CMR has emerged as a noninvasive cornerstone [1]. In our case, the constellation of clinical features, viral positivity with response to specific antiviral therapy, and imaging findings, strongly favored the diagnosis, obviating the need for biopsy.

The pathophysiological evolution of fulminant myocarditis often culminates in cardiogenic shock, defined by tissue hypoperfusion and end‐organ dysfunction despite adequate preload. According to the SCAI classification, our patient progressed rapidly from Stage C to E shock, culminating in refractory cardiac arrest [5]. This trajectory underscores the unpredictability of fulminant myocarditis and the necessity for immediate escalation to advanced MCS when pharmacological therapy fails.

Among available MCS devices, the Impella CP microaxial pump was initially deployed, providing left ventricular unloading, reduction in wall stress, and augmentation of coronary perfusion. In fulminant myocarditis, these physiological effects are particularly advantageous, as they may attenuate the inflammatory cascade and limit irreversible injury [6]. Observational studies and registry analyses have reported favorable survival rates with Impella support in myocarditis, especially in younger patients, although complications such as bleeding and renal injury remain nonnegligible [1, 2]. Nevertheless, the use of Impella in fulminant myocarditis remains largely guided by expert consensus and case‐series data, with randomized controlled evidence still lacking [2].

Despite Impella CP support, the patient′s condition deteriorated into refractory cardiac arrest. In this setting, VA‐ECMO was instituted as ECPR. The dual role of VA‐ECMO in fulminant myocarditis encompasses both systemic perfusion support and, when combined with Impella (“ECMELLA” configuration), ventricular unloading. Literature demonstrates survival rates ranging from 40% to 88% in fulminant myocarditis patients managed with VA‐ECMO, substantially higher than survival in other etiologies of cardiogenic shock, reflecting the reversibility of viral myocarditis [7, 8]. Favorable clinical outcomes have also been observed with combined MCS using ECMELLA approach, with a reported survival rate of 68.5%. However, these results were accompanied by a substantial overall complication rate of 48.7%, predominantly driven by bleeding events (32%) and acute kidney injury (8.6%) [9].

Prognosis is strongly time‐dependent: Shorter “low‐flow” intervals and rapid VA‐ECMO deployment are associated with improved neurological outcomes [10]. In this case, immediate initiation of automated chest compressions using the LUCAS 2 device allowed safe patient transfer and uninterrupted high‐quality cardiopulmonary resuscitation until VA‐ECMO implantation, contributing to the excellent neurological recovery.

The role of automated mechanical chest compression devices remains debated. Large randomized trials in out‐of‐hospital cardiac arrest have not consistently demonstrated survival benefits, yet such devices are valuable in scenarios where manual compressions are impractical—such as during transport or invasive procedures [11–14]. Nevertheless, the use of automated chest compression devices is associated with complications, mainly skeletal and visceral injuries with a potentially increased risk of hemorrhage, including hemothorax and pulmonary contusion. Accordingly, current international guidelines do not recommend their routine use [15]. In our experience, automated mechanical chest compressions proved feasible and safe as a bridge to VA‐ECMO implantation, particularly in settings where regional organizational constraints precluded the activation of an on‐site mobile ECMO team.

The subsequent clinical course was notable for complete recovery of biventricular function, confirmed by echocardiography and CMR, and sustained at 6‐month follow‐up. This outcome highlights the reversibility of fulminant myocarditis when timely MCS prevents irreversible organ damage. Moreover, it illustrates the therapeutic window in which aggressive hemodynamic stabilization and circulatory support may allow native myocardial recovery, preventing the need for durable left ventricular assist device implantation or transplantation.

This case also raises broader considerations regarding patient selection and timing of MCS in fulminant myocarditis. Although no consensus guidelines exist, several prognostic tools such as Survival After Venoarterial Extracorporeal Membrane Oxygenation (SAVE score) and SCAI classification may aid in stratification. Nevertheless, decisions must be individualized, taking into account comorbidities, prearrest organ function, and local expertise. Importantly, survival in fulminant myocarditis is contingent not only on device deployment but also on system‐level factors, including regional organization of ECMO networks, availability of trained personnel, and rapid triage protocols [2].

## 4. Conclusion

Fulminant myocarditis due to Influenza A (H1N1) remains a life‐threatening but potentially reversible condition. Early recognition, prompt exclusion of coronary artery disease, and rapid escalation to MCS are crucial determinants of outcome. The combined use of Impella and VA‐ECMO, facilitated by automated chest compression devices, enabled full recovery in this patient. This case underscores the importance of integrated multidisciplinary management and organizational preparedness in optimizing outcomes for fulminant myocarditis.

## Funding

No funding was received for this manuscript.

## Disclosure

All authors have read and approved the final version of the manuscript. The corresponding author had full access to all of the data in this study and takes complete responsibility for the integrity of the data and the accuracy of the data analysis. The manuscript guarantor affirms that this manuscript is an honest, accurate, and transparent account of the case being reported; that no important aspects of the case have been omitted; and that all relevant clinical information has been reported accurately.

## Ethics Statement

The study was conducted in accordance with the principles of the Declaration of Helsinki.

Formal approval by the local Ethics Committee was not required for this single‐patient case report according to institutional policy. Written informed consent for publication of the clinical information and associated images was obtained from the patient.

## Consent

Written informed consent was obtained from the patient for publication of this case report and any accompanying images.

## Conflicts of Interest

The authors declare no conflicts of interest.

## Data Availability

The data supporting the findings of this study are available from the corresponding author upon reasonable request. Due to privacy and ethical restrictions, patient‐level data are not publicly available.
